# The Role of Affect and Cognition in Processing Messages about Early Diagnosis for Alzheimer’s Disease by Older People

**DOI:** 10.3390/healthcare5020027

**Published:** 2017-06-12

**Authors:** Patrick De Pelsmacker, Martine Lewi, Veroline Cauberghe

**Affiliations:** 1 Faculty of Applied Economics, University of Antwerp, Prinsstraat 13, 2000 Antwerpen, Belgium; tine.lewi@gmail.com; 2 Department of Communication Sciences, Ghent University, Korte Meer 7-11, 9000 Gent, Belgium; veroline.cauberghe@ugent.be

**Keywords:** Alzheimer’s disease, early diagnosis, message argument strength, message image valence, affect, thoughts, population at risk

## Abstract

Through early diagnosis of symptoms, the Alzheimer’s disease process can be decelerated. The main concern is to encourage the population at risk to take responsible actions at the earliest stage of the onset of the disease. Persuasive communication is essential to achieve this. In an experimental study, the evaluation of awareness messages for early diagnosis containing weak and strong arguments and negative and positive images was performed on a sample of older Belgians. The mediating role of affective responses and message thoughts was explored. Strong arguments led to a more positive evaluation of the message than weak arguments directly and indirectly via the positive effect they had on message affect and thoughts, which, in turn, positively affected message evaluation. A negative message image led to a more positive message evaluation than a positive one. This effect was not mediated by either message affect or message thoughts.

## 1. Introduction

Alzheimer’s disease is a devastating, incurable disease. The disease pathway is typically characterized by a long preclinical phase in which patients experience mild cognitive impairments, such as forgetting recent events or repeating the same question, struggling with thinking things through, being very easily distracted, taking much longer than usual to find the right word for something, or struggling to interpret an object in three dimensions, judge distances or navigate stairs [1]. Through early diagnosis of symptoms, the disease process can be decelerated [2,3]. The main concern is then to make the population at risk aware of the importance of early diagnosis and stimulate them to take responsible actions at the earliest stage of the onset of the disease. Many studies have been published that test the effect of awareness campaigns to promote early prevention and diagnosis of several diseases, such as breast cancer [4,5,6] and colorectal cancer [7], and the effects of various communication strategies, such as message personalization [4,8], message framing [5,9], and message endorsement [7]. However, very little is known about the efficacy of health communication in the domain of a disease such as Alzheimer’s, where one can currently merely intervene in order to lengthen the time period in which the patient can still enjoy a reasonable quality of life.

The present study aims to explore the role of evoked message affect and thoughts about the message as mediators in the processing of Alzheimer’s awareness messages varying in argument strength and valence (positive or negative image). We study the extent to which these factors have an impact on the evaluation of the message. The study investigates older people, an age cohort that is a prime target group for Alzheimer’s awareness messages. To that end, we carried out an experiment with 245 Belgian participants.

Strong arguments led to a more positive evaluation of the message than weak arguments directly and indirectly via the positive effect they had on message affect and thoughts, which in turn, positively affected message evaluation. A negative message image led to a more positive message evaluation than did a positive image. This effect was not mediated by either message affect or message thoughts.

The study adds to our understanding of how differently framed health messages regarding early diagnosis of a fatal illness are processed by the prime target group, and the role of affective and cognitive message responses in this process. The study also informs health communication practitioners and educators on how to design their awareness messages for early diagnosis of Alzheimer’s disease.

## 2. Conceptual Framework and Research Questions

**Figure 1:**
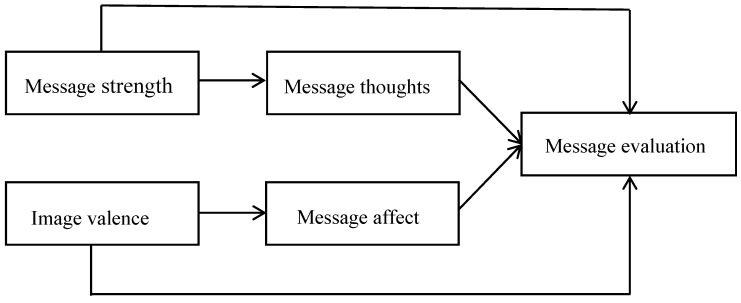
Conceptual model.

The conceptual framework reflects the purpose of this study (Figure 1): The effects of message strength and image valence on message evaluation, as mediated by the valence of the evoked message thoughts and message affect, was considered.

### 2.1. Argument Strength and Image Valence

Awareness messages can contain arguments varying in strength. Prior communication research shows that strong argument messages are usually evaluated more positively [10]; examples include research related to charity and fundraising [11], health communication settings [12], and smoking cessation [13,14]. In general, using visuals or images with a certain valence, can have a significant effect on the viewer’s evaluation of a message [15]. Prior research even provides support for a “visual dominance” effect in information processing in that visual cues are more salient, attention-grabbing, and persuasive than printed words [16,17,18,19]. Images can also influence adherence to health instructions [20]. However, previous research is inconclusive as to the effect of the valence of a visual or an image (positive or negative) on message evaluation. Some previous studies point at a positive effect of positively valenced message images. For instance, in the area of smoking prevention or cessation, messages accompanied by positive images had a more persuasive effect than those accompanied by negative fear-arousing images [14]. Moreover, in anti-alcohol campaigns, distressing images inhibit persuasion [21]. On the other hand, for instance, in the context of accident prevention, Jones, Chapman, and Bailey [22] found that participants show a higher degree of awareness of potential hazards when a neutral image was shown, in comparison to a positively and a negatively valenced image.

### 2.2. Cognitive Responses

According to the Elaboration Likelihood model (ELM), message arguments may be processed in two different ways. In central processing, message-evoked thoughts may lead to a central evaluation of the message, leading to a profound change in attitude. Alternatively, messages may be processed peripherally, in which case cognitive responses are less instrumental for processing the message, and attitudes are only superficially changed [23]. Awareness messages can evoke negative and positive thoughts. These may be triggered by both textual arguments and visuals in a message. Indeed, the Alzheimer’s issue may for many older people be a serious concern, and they therefore may be motivated to process the arguments in an Alzheimer’s awareness message centrally [24,25,26]. Consequently, processing may be expected in which message-evoked thoughts substantially mediate the positive or negative effect of message arguments on the evaluation of the message. On the other hand, prior research shows that a message image can also change a consumer’s cognitive activity while viewing the ad [15]. Indeed, cognitive elaboration may be a necessary condition to produce a positive or negative effect of message vividness (by means of using images) on attitudes towards the message [27]. Since it is unclear the extent to which message strength and message image valence leads to cognitive processing, and further influence message evaluation, we formulated the following research question:

***RQ1.***  Does the cognitive response to the message (the valence of the thoughts about the Alzheimer’s disease awareness message) mediate the effect of (a) the strength of the arguments and (b) the affective valence of the visual on the evaluation of an Alzheimer’s awareness message?

### 2.3. Affective Responses

Research has developed and tested theories of judgment and decision making that incorporate affect as a key component in a process of constructing values and preferences. Affect refers to positive and negative feelings about a message that are generally based on prior experiences, and are experienced while being exposed to a message stimulus. Affect can act as a spotlight focusing our attention, it can act as information, and it can motivate action or the processing of information [28]. According to Forgas’ Affect Infusion Model (AIM) [29,30], two underlying mechanisms of affect infusion are affect-as-information and affect priming. The affect-as-information theory suggests that individuals may ask themselves: “How do I feel about the message?” and in doing so, may be guided by their feelings to judge a message [30,31]. In affect priming, affect can inform judgments by facilitating access to related cognitive categories [32,33,34]. It is implied that it is in the course of substantive, constructive processing affect is most likely to play a significant role in how message information is interpreted. Stimuli that are perceived as positively affective should lead to making more positive interpretations of information [29]. Several studies have found significant affective processing effects in judgments about persuasive messages, mostly under conditions of substantive processing (judgments about more complex stimuli) [20,35]. Applied to the domain of health, Salovey and Birnbaum [36] found that affective reactions had a major influence on people’s perceptions of symptoms of illness, their health efficacy judgments, and their expectations of future disease. Salovey et al. [37] concluded that these effects are due to affect-priming mechanisms operating during substantive processing.

Both verbal (arguments) and visual (images) message elements may trigger affective responses [20,29]. It is therefore expected that affective responses to messages mediate the positive or negative effect of verbal and visual message components on the evaluation of a message. Since it is unclear the extent to which message strength and message image valence will lead to affective processing, and further influence the evaluation of the message, we formulated the following research question:

***RQ2.***  Does the affective response to an Alzheimer’s disease awareness message mediate the effect of (a) the strength of the arguments and (b) the affective valence of the visual on the evaluation of an Alzheimer’s awareness message?

## 3. Materials and Methods

### 3.1. Research Design and Stimuli

A 2 (argument strength: weak—strong) × 2 (affective valence of image: negative—positive) between subjects experimental design was used to collect the data. Based on a series of pre-tests involving physicians, general public and caregivers, two sets of arguments and two pictures were selected. Based on a *t*-test, the two images differed significantly (*p* < 0.05) on affective valence (three-item, 7-point Likert scale): negative image (M = 3.44), positive image (M = 5.88). The two message texts differed significantly (*t*-test, *p* < 0.05) on message strength (three-item, 7-point Likert scale): weak message (M = 4.23) and strong message (M = 5.68). All messages communicate the benefits of early diagnosis of Alzheimer’s disease. The resulting two images and two texts were then combined into four Alzheimer’s disease awareness messages and used in the main study (see Appendix A for examples).

### 3.2. Procedure and Sample

An online survey was conducted in a sample of 245 Belgian individuals between 55 and 65 years old (49% females and 51% males). Respondents were recruited by a professional research company. They received an e-mail with a link to a web survey. At the time of the study, and after considering the nature and scope of the study, the university’s institutional review board did not consider it necessary to enter a formal approval procedure. After the respondents opened the online survey (clicking the link), they were fully informed about the nature and purpose of the study, that they would participate in a fully anonymous manner, and that the data would be consequently analyzed in a fully anonymous way. They were also informed that they could withdraw from the study at any time. After answering a number of demographic questions, they viewed one of the four Alzheimer’s disease messages, to which they were randomly assigned. Subsequently, they answered questions on scales evaluating their responses to the message.

### 3.3. Measures

The two independent variables, argument strength and image valence, are manipulated in the experiment. The two mediators are perceived message affect and thoughts about the message. Message affect was measured on a 5-item 7-point scale (happy—sad, hopeful—desperate, assured—worried, warm-hearted—cold-hearted, secure—unsafe) (α = 0.805) [38]. As for message thoughts, after exposure to the message, participants were asked to freely elicit their thoughts about the message. All thoughts were coded according to their valence (negative, neutral, positive) by two independent judges. They agreed in 94% of the cases. Disagreement was resolved by a third judge. The variable “message thoughts” used in the analyses was defined as the sum of the number of positive (+1), negative (−1), and neutral (0) thoughts.

The dependent variable is Evaluation—“evaluation of the message”. It was measured through a 3-item 7-point scale (I find the health communication strong/weak; this communication makes a good/bad impression on me; I like/dislike this communication) (α = 0.915) [32]. 

## 4. Results

The analyses were conducted using Hayes’ PROCESS Model 4 [39]. Hayes’ model has become one of the standard computational tools for the analysis of mediation and moderation in the behavioral sciences. It is a versatile analytical instrument that allows analysis of observed mediation, moderation, and conditional effects. In these analyses, argument strength and image valence are the independent variables, message affect and message thoughts are the mediators, and message evaluation is the outcome variable. Two separate models were run, one for each of the independent variables, with the two mediators included in every model.

In the first model (full statistical details in Table 1), mediation was investigated for the effect of argument strength (weak/strong argument message). There is a direct effect of argument strength on message evaluation (b = 0.546, *p* = 0.001). Participants who viewed the strong message reported a more positive message evaluation. Message thoughts partially mediated the effect of message strength on message evaluation (indirect effect: b = 0.169, *p* = 0.007) (RQ1a). A strong message led to more positive message thoughts. Further, more positive message thoughts led to a more positive message evaluation. Message affect also partially mediated the effects of message strength on message evaluation (b = 0.224, *p* = 0.013) (RQ2a). A strong message led to more positive message affect, and more positive message affect led to a more positive message evaluation. The second independent variable that was investigated was message image valence—comparing negative versus positive valenced images (full statistical details in Table 2). There was a direct negative effect of image valence on message evaluation (b = −0.452, *p* = 0.017). A negative image led to a more positive message evaluation. Neither message thoughts (indirect effect: b = 0.884, *p* = 0.377) nor message affect (indirect effect: b = 0.108, *p* = 0.427) mediated the effect of image valence on message evaluation (RQ1b and RQ2b).

## 5. Discussion

Strong arguments led to a more positive evaluation of the message than did weak arguments. This outcome is in line with prior research [10,11,12,13,14]. Positive message thoughts and positive affective responses to the message led to a more positive message evaluation, confirming prior findings [20,24,25,26,35,36,37]. Message thoughts and message affect significantly mediated the effect of message strength on message evaluation in that stronger arguments led to more positive message thoughts and feelings, which, in turn, led to a more positive message evaluation. This finding indicates that, for the observed group of older people, argument strength is a factor that triggers both positive affective and cognitive elaboration, leading to more message appreciation. This is in line with earlier findings [24,25,26,28,29,30,31,36,37]. Interestingly, a negative message image led to a more positive message evaluation than did a positive image. The fact that positively valenced images do not necessarily trigger positive evaluative message responses is in line with previous findings [16]. The effect of image valence on message evaluation was not mediated by either message thoughts or message affect. This result is not in line with earlier findings that a dominant image in a print ad can change viewers’ cognitive activity while viewing the message [9,27] and that cognitive elaboration could be a necessary condition to produce an effect for vividness on attitudes [20,29], nor with the affect priming principle [29,30,32].

Insights into these processing mechanisms are important for the development of tailor-made, and hence potentially more persuasive, awareness messages. The older public investigated in the current study, which is a prime target group of Alzheimer’s awareness messages, valued messages with strong arguments more than they did ones with weak arguments. They also evaluated a message more positively when the messages evoked positive affective responses. To appeal to them, it was also important that message arguments trigger positive message thoughts. It is advised that images that present Alzheimer’s disease as a positive thing be avoided, since they trigger negative message evaluation.

In the current study, the effect of argument strength and image valence was investigated separately because the analytical method we used to test the mediation effects only allowed for one independent variable. Further research could investigate interaction effects between the two variables to explore the extent to which the two main effects of argument strength and image valence are further qualified by the effect of an interaction between them. Additionally, the interaction between either argument strength or image valence on the one hand, and other message framing factors on the other, could be further investigated. For instance, prior research demonstrated that positively framed messages (messages that emphasize the positive consequences of certain actions) combined with positive images had a more persuasive effect compared to negatively framed messages combined with negative fear-arousing images [14,21]. The exploration of these potential interaction effects is a promising angle for further research. Additionally, the role of endorsers may be studied: Does it make a difference for message responses whether a message is brought by a medical expert (medical doctor), a patient suffering from Alzheimer’s disease, or a caregiver?

Other format elements that could evoke cognitive and affective reactions should be studied, such as message framing (loss or gain framing), the type of information provided, and the nature of the images used. Personal characteristics and how these play a role in processing persuasive communication, such as the level of experience, risk perception, and health awareness, should be studied as well [40,41,42].

The current approach to awareness messages could also be tested for different health issues, for example, in the area of cancer screening [43]. Investigating the impact of message components and affective and cognitive responses to messages on various aspects of behavioral intention and behavior itself, such as a visit to a doctor, a search for more information, and vigilance about early warning signals, is yet another avenue for further research.

Finally, different people may have different message processing styles. For instance, individuals could be generally inclined to process messages centrally or peripherally. Taking individual differences between individuals into account to assess their response to awareness messages is a promising avenue for further research.

## 6. Conclusions

For making older people aware of the importance of early diagnosis of Alzheimer’s disease, strong arguments lead to a more positive evaluation of the message than weak ones. Message thoughts and message affect significantly mediate the effect of message strength on message evaluation in that stronger arguments led to more positive message thoughts and feelings, which, in turn, led to a more positive message evaluation. Interestingly, a negative message image leads to a more positive message evaluation than a positive image. The effect of image valence on message evaluation was not mediated by either message thoughts or message affect. To appeal to older people it is thus important strong message arguments trigger positive message thoughts and feelings. Images that present Alzheimer’s disease as a positive thing should be avoided.

## Figures and Tables

**Table 1:** Model estimation results with message strength as the independent variable.

Dependent	Independent	Coeff.	SE	t	*p*	LLCI	ULCI
Message affect	Constant	4.215	0.0923	45.376	<0.001	4.032	4.398
	Argument strength						
Message thoughts	Constant	0.208	0.200	1.044	0.298	−0.185	0.602
	Argument strength	0.888	0.280	3.174	0.002	0.337	1.440
Message evaluation	Constant	1.386	0.337	4.112	<0.001	0.722	2.049
	Message affect	0.662	0.076	8.727	<0.001	0.513	0.812
	Message thoughts	0.190	0.035	5.386	<0.001	0.121	0.260
	Argument strength	0.546	0.157	3.478	<0.001	0.237	0.856
**Indirect effect of Argument strength**	**Effect**	**SE**	**Z**	***p***			
Through Message affect	0.224	0.091	2.472	0.013			
Through Message thoughts	0.169	0.063	2.700	0.007			

Note: LLCI = lower limit of 95% confidence interval; ULCI = higher limit of P5% confidence interval of effect.

**Table 2:** Model estimation results with image valence as the independent variable.

Dependent	Independent	Coeff.	SE	t	*p*	LLCI	ULCI
Message affect	Constant	4.247	0.114	37.353	<0.001	4.023	4.472
	Image valence	0.131	0.163	0.803	0.423	−0.191	0.452
Message thoughts	Constant	0.518	0.243	2.129	0.035	0.038	0.998
	Image valence	0.322	0.348	0.925	0.357	−0.365	1.009
Message evaluation	Constant	1.277	0.405	3.156	0.002	0.478	2.075
	Message affect	0.801	0.091	8.823	<0.001	0.622	0.980
	Message thoughts	0.189	0.043	4.446	<0.001	0.105	0.273
	Image valence	−0.452	0.188	−2.409	0.017	−0.823	−0.082
**Indirect effect of Image valence**	**Effect**	**SE**	**Z**	***p***			
Through Message affect	0.105	0.132	0.795	0.427			
Through Message thoughts	0.061	0.069	0.884	0.377			

Note: LLCI = lower limit of 95% confidence interval; ULCI = higher limit of P5% confidence interval of effect.

**Figure A1:**
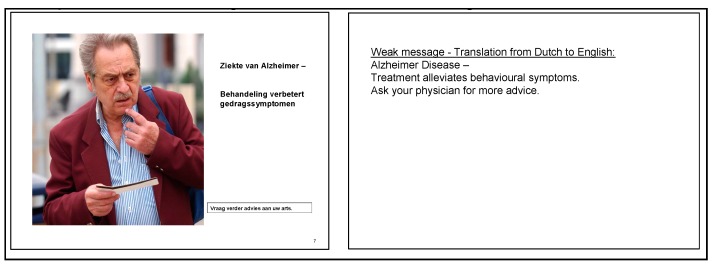
Example of combination negative affect with weak message.

**Figure A2:**
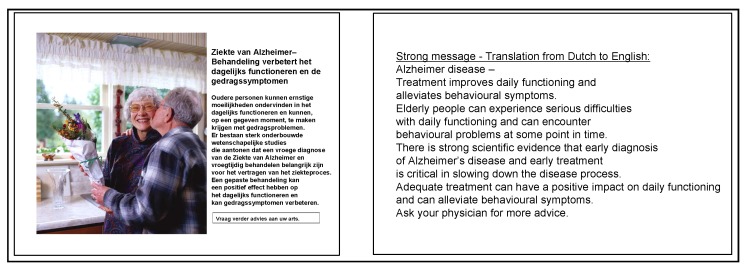
Example of combination positive affect with strong message.
